# Spontaneous splenic rupture as the first manifestation of chronic myeloid leukemia: A case report

**DOI:** 10.5339/qmj.2026.47

**Published:** 2026-06-23

**Authors:** Albaraa Rustom, Zeinab Ibrahim, Yaman Alahmad, Omar Jamil, Rayan Mahgoub, Salman Mirza

**Affiliations:** 1 Department of Clinical Imaging, Hamad Medical Corporation, Doha, Qatar

**Keywords:** Splenic rupture, leukemia, myelogenous, chronic, splenectomy, Qatar

## Abstract

**Background:**

Spontaneous splenic rupture (SSR) is an uncommon life-threatening complication of chronic myeloid leukemia (CML); however, it rarely occurs as the initial presentation of the disease. A contrast-enhanced computed tomography (CT) scan is the preferred method for confirming the diagnosis. The definitive treatment is radical splenectomy.

**Case Presentation:**

We report the case of a 37-year-old previously healthy man who presented to the Emergency Department with severe, vague abdominal pain and nausea of one day’s duration. On physical examination, he had epigastric tenderness and palpable splenomegaly, while his vital signs were within the normal limits. Contrast-enhanced CT revealed massive splenomegaly and splenic rupture with active bleeding. Subsequently, the patient became hemodynamically unstable and required an urgent total splenectomy. Molecular cytogenetic tests showed abnormalities consistent with CML. The patient’s condition gradually improved, and he was discharged home on the 10th postoperative day.

**Discussion:**

SSR typically occurs in patients with preexisting hematologic disorders or constitutional symptoms. This case is unique because the patient, who had no history of trauma, constitutional symptoms, or a known hematologic disorder, presented with massive splenomegaly and extremely elevated leukocytosis and was found to have undiagnosed CML. The patient’s massive splenomegaly and abrupt splenic rupture highlight that SSR can occur even in the absence of classic B symptoms, posing significant diagnostic and management challenges. Timely multidisciplinary intervention is crucial, and thorough post-splenectomy care is essential.

**Conclusion:**

SSR as the initial manifestation of CML is an uncommon and unexpected clinical presentation. This case demonstrates that extreme leukocytosis and massive splenomegaly can precipitate splenic rupture in the absence of prodromal symptoms. Timely surgical intervention, comprehensive post-splenectomy care, and the prompt initiation of targeted therapy are indispensable for achieving optimal clinical outcomes, as demonstrated in our patient.

## 1. INTRODUCTION

Spontaneous splenic rupture (SSR) is an uncommon yet potentially fatal event that occurs in the absence of trauma. It is frequently linked to infections, hematologic malignancies, and certain pharmacologic agents. Due to its rich vascular network, the spleen is particularly susceptible to rupture as a result of massive infiltration, splenomegaly-induced pressure, or compromised vascular integrity.^1^

Chronic myeloid leukemia (CML) is a type of myeloproliferative neoplasm that accounts for approximately 15% of adult leukemia cases and occurs at an annual incidence of about two cases per 100,000 individuals.^2^ The disease is defined by a specific chromosomal abnormality, t(9;22)(q34;q11.2), which results in the fusion of the *ABL1* gene from chromosome 9 with the *BCR* gene on chromosome 22. This translocation produces the Philadelphia chromosome and leads to the formation of the *BCR::ABL1* fusion gene. The resulting oncoprotein drives continuous tyrosine kinase activity, which in turn promotes uncontrolled proliferation of myeloid cells and disrupts normal hematopoiesis.^2^

Hematologic evaluation is essential for identifying the underlying causes, such as CML. Bone marrow studies are crucial for diagnosing and staging CML. They typically show hypercellularity with granulocytic expansion and fewer than 10% blasts in the chronic phase. Cytogenetics confirms the presence of the Philadelphia chromosome, while molecular tests detect the *BCR::ABL1* fusion gene.^3^ Notably, recent research suggests that changes in the bone marrow niche, such as decreased CXCL14 expression, can support leukemic cell survival and contribute to therapeutic resistance.^4^

This is a unique case of an atypical presentation of CML, characterized by an extremely elevated leukocyte count occurring in the absence of constitutional symptoms, trauma, or a prior hematologic disorder.

This case was managed at Hamad General Hospital, a modern tertiary care facility within Hamad Medical Corporation (HMC), the principal public provider of secondary and tertiary healthcare in the State of Qatar.^5^ Written consent was obtained from the patient, and the Medical Research Center (MRC) granted approval for the publication of this case report (MRC-04-25-1181).

## 2. CASE PRESENTATION

A 37-year-old gentleman with no known past medical history presented to the general Emergency Department in May 2025, with acute, severe left-sided abdominal pain for one day, which had shifted to the right lower abdomen in the last few hours. The pain was associated with nausea; however, the patient denied fever, vomiting, urinary symptoms, or any change in bowel habits. He was not taking any medication, and all the vital signs were within the normal limits. The Glasgow Coma Scale was 15, the oral temperature was 37.2°C (normal range: 35.5–37.9°C), and the documented body mass index was 22.5 kg/m^2^ (normal range: 18.5–24.9 kg/m^2^).

Physical examination revealed a soft abdomen with tenderness in the epigastrium and the left abdomen, along with palpable splenomegaly.

Laboratory values obtained upon presentation to the Emergency Department and on admission are listed in Table 1.

**Figure 1.**
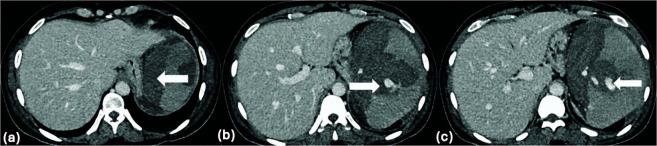
Axial images of the CT scan of the abdomen with IV contrast in venous phase: (a) subcapsular hematoma (white arrow); (b) and (c) multiple foci of active IV contrast extravasation (white arrow), suggestive of active bleeding, in addition to the splenic laceration and hematoma.

**Figure 2.**
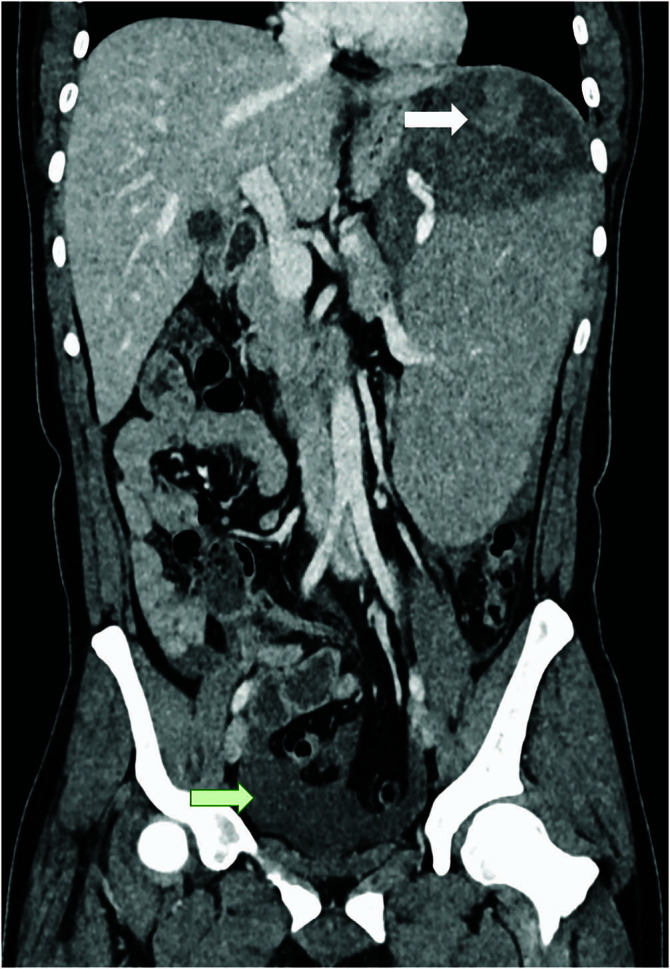
Coronal image of the CT scan of the abdomen with IV contrast in the venous phase showing a massively enlarged spleen measuring approximately 23 cm in craniocaudal dimension and an upper-pole splenic laceration (white arrow). Additionally, a mild-to-moderate hemoperitoneum can also be seen (green arrow).

While in the Emergency Department, a contrast-enhanced CT scan of the abdomen was performed. It showed a massively enlarged spleen measuring approximately 23 cm in craniocaudal dimension, with an upper-pole splenic laceration, and multiple foci of active intravenous (IV) contrast extravasation suggestive of active bleeding. In addition to a large peri-splenic subcapsular hematoma, a mild-to-moderate hemoperitoneum was also noted (Figures 1 and 2).

Following the CT scan, follow-up blood tests were requested to assess the hemoglobin level. Repeated laboratory investigations revealed an acute drop in the hemoglobin level (91 g/L), as indicated in the admission column in Table 1. The patient was initially evaluated by the interventional radiology team for possible splenic artery embolization. However, following a multidisciplinary discussion involving interventional radiology, the acute care surgery team, emergency physicians, and the on-call hematologist, the patient’s hemodynamic instability necessitated immediate emergent laparotomy and splenectomy.

The patient underwent emergency surgery via a midline laparotomy under general anesthesia. Intraoperative findings demonstrated massive splenomegaly with ruptured capsule and blood clots all over the peritoneal cavity. A splenectomy with ligation of the splenic artery and vein was performed, and general assessment of the peritoneum showed no other concerning findings. During the surgery, the patient was transfused with two units of packed red blood cells. The procedure was well tolerated, with no intraoperative complications. Postoperatively, the patient recovered uneventfully from anesthesia and was extubated without difficulty. He remained hemodynamically stable, with no immediate postoperative complications. The patient was transferred to the surgical ward in stable condition.

**Figure 3.**
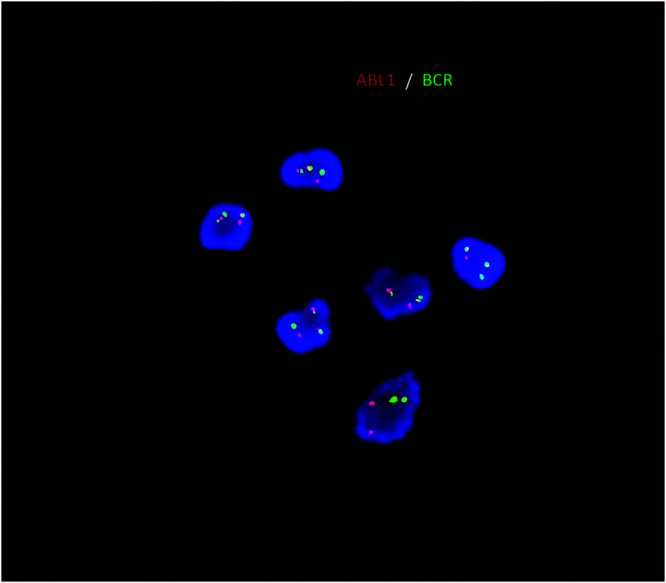
iFISH evaluation of bone marrow interphase nuclei showing the BCR:ABL1 rearrangement.

**Figure 4.**
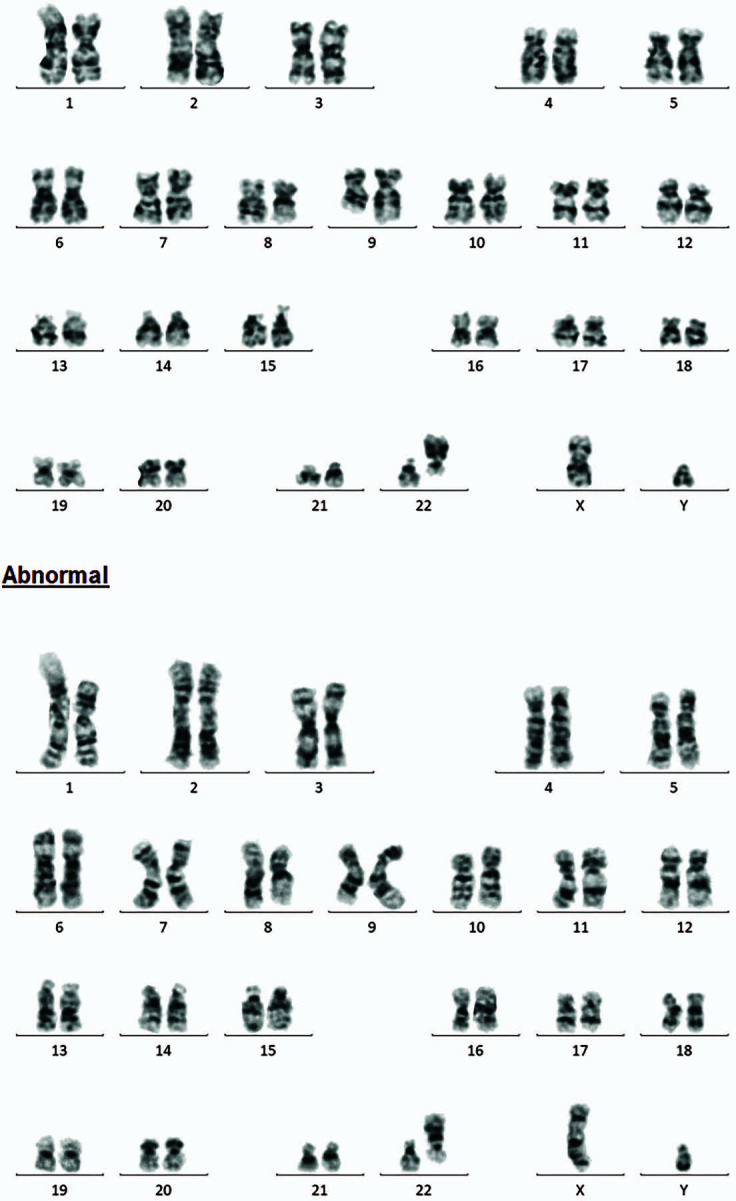
G-banding karyotype analysis of bone marrow demonstrating a complex variant of the Philadelphia chromosome involving chromosomes 1, 8, 9, and 22.

**Figure 5.**
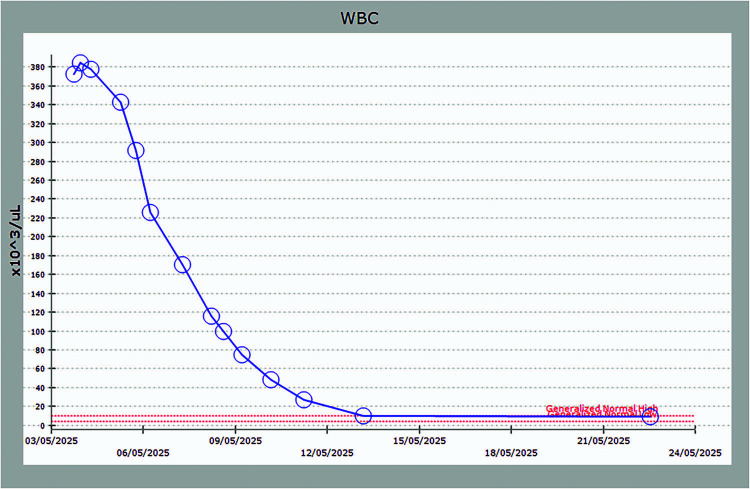
Serum leukocyte count trends following treatment initiation.

During the patient’s hospital stay, the consulting hematologist requested a peripheral blood smear, which showed features suggestive of CML, including hyperleukocytosis with a shift to the left, normocytic normochromic anemia, basophilia, eosinophilia, and normal platelet count. Based on these findings, the hematology team initiated chemotherapy with oral hydroxyurea at a dose of 1,000 mg twice daily, in addition to oral allopurinol 300 mg once daily, with no adverse effects noted. For confirmation of the diagnosis, the hematologist requested an interphase fluorescence *in situ* hybridization (iFISH) test, which demonstrated a BCR::ABL1 fusion (Figure 3). Chromosomal analysis was also performed, which revealed the Philadelphia chromosome (Figure 4). The diagnosis of CML was established. The patient showed improvement on the current treatment plan, and his leukocyte count showed a gradual downward trend (Figure 5). With signs of improvement, the hydroxyurea dose was reduced to 500 mg twice daily. Repeated blood test after two days showed a normal leukocyte count (9.8×10^9^/L); therefore, hydroxyurea was discontinued.

The case was discussed in the hematology multidisciplinary team (MDT) meeting to determine the plan of care. The MDT recommended initiation of a second-generation tyrosine kinase inhibitor (TKI) and monitoring of *BCR::ABL1* levels.

The patient was clinically stable and fit for discharge, with a normal leukocyte count and no active complaints. He received pre-discharge immunization against *Streptococcus pneumoniae*, *Hemophilus influenzae* type b, and *Neisseria meningitidis*. Upon discharge, the patient was prescribed oral dasatinib, a second-generation TKI, at a dose of 100 mg once daily, as per MDT recommendations, and he agreed to the treatment plan. He was discharged with close follow-up in the hematology clinic, along with counseling to seek prompt medical attention for febrile illnesses.

At the time of submission of this case report, three months post-discharge, the patient was regularly attending his face-to-face appointments in the hematology clinic. He was doing well on dasatinib tablets alone, and follow-up laboratory tests showed a normal leukocyte count, with *BCR::ABL1* levels within the desired range.

## 3. DISCUSSION

SSR is a surgical emergency that is rarely the initial presentation of CML. Splenomegaly is common in CML; however, progression to rupture without trauma or coexistent infection is exceptional and poses both diagnostic and management challenges.^2,6^

In radiologic evaluation, contrast-enhanced CT is the cornerstone imaging modality for diagnosing SSR, particularly in the absence of trauma. It offers high-resolution visualization of the splenic parenchyma, capsular integrity, peri-splenic fluid, and active contrast extravasation—features essential for confirming rupture and assessing the extent of hemorrhage.^3^

This case presentation stands out for several reasons: the patient had no constitutional symptoms, no history of trauma, and no prior hematologic diagnosis, yet he exhibited an extraordinarily elevated leukocyte count (>370×10^9^/L). Such extreme leukocytosis likely reflects rapid leukemic infiltration of the spleen, leading to parenchymal congestion, capsular tension, and vascular compromise. Although these mechanisms have been documented in prior reviews, they are rarely correlated with such profound leukocytosis, which likely precipitated his splenic rupture.^7,8^ Furthermore, the massive splenic size (≈23 cm) exceeded the splenic dimensions typically reported in similar cases, which usually range between 15 and 20 cm,^1,9^ suggesting a more aggressive disease burden despite the absence of systemic symptoms.

In contrast to our patient, the existing reports describe SSR in CML typically occurring in patients with constitutional symptoms, splenomegaly-related discomfort, or previously known hematologic disorders. For example, Zioui et al.^6^ reported hemorrhagic shock with relatively modest leukocytosis (~150×10^9^/L) in a patient with constitutional symptoms and a history of pulmonary tuberculosis, while Dahake et al.^7^ reported splenic rupture after months of splenomegaly-related discomfort accompanied by multiple nonspecific systematic symptoms and dark tarry stool. Our patient’s unusual presentation of vague migratory abdominal pain, stable initial vital signs, and extreme leukocytosis reflects an atypical and clinically deceptive presentation. This highlights that the absence of typical B symptoms does not exclude catastrophic splenic pathology and adds a new profile to the spectrum of SSR in CML. In a systematic review of 613 cases, Aubrey-Bassler et al.^9^ found that SSR may present as the first manifestation of an underlying disease, which may be either very subtle or completely unapparent in the history of present illness, and that it frequently occurs in patients without prior splenic pathology or trauma. This underscores the unpredictability and the diagnostic challenges of such events. Consequently, maintaining a low threshold for multidisciplinary discussion (emergency medicine, surgery, interventional radiology, and hematology) can expedite life-saving intervention and prevent devastating complications.^10^

From a therapeutic standpoint, most reported cases required urgent splenectomy followed by delayed initiation of chemotherapy or TKIs once the diagnosis was established. In our case, early initiation of chemotherapy with hydroxyurea resulted in rapid hematologic improvement, allowing a timely transition to second-generation TKI therapy (dasatinib). Unlike some previously reported cases with limited follow-up data or poor outcomes related to hemodynamic instability, our patient demonstrated favorable short- and long-term outcomes, with normalization of leukocyte counts and BCR::ABL1 levels within the desired therapeutic range on follow-up.^11,12^ It should be noted that splenectomy mandates lifelong vigilance for encapsulated bacterial infection. In summary, SSR can be the first herald of a silent hematologic malignancy, and clinicians should remain alert to this possibility.^13^

## 4. CONCLUSION

SSR as an initial manifestation of CML is an extraordinary clinical encounter. This case demonstrates that extreme leukocytosis and splenomegaly can precipitate rupture without prodromal symptoms, and that decisive surgical intervention, comprehensive post-splenectomy care, and rapid initiation of targeted therapy are essential to achieving favorable outcomes.

## DATA AVAILABILITY

All data relevant to this case report are included in this article.

## AUTHORS’ CONTRIBUTIONS

All authors contributed equally and read and approved the final manuscript.

## FUNDING

No funds, grants, or other support were received.

## ACKNOWLEDGMENTS

The authors have no acknowledgements to declare.

## DISCLOSURE OF AI USE

No generative AI or AI-assisted tools were used in writing this case report.

## CONSENT TO PARTICIPATE

Written informed consent for the preparation and publication of this case report was obtained from the patient.

## COMPETING INTERESTS

The authors have no relevant financial or non-financial interests to disclose.

## Figures and Tables

**Table 1.** Laboratory parameters on presentation to the Emergency Department (ED) and 5 h later at the time of in-patient admission.

Parameter	Normal values	On ED presentation	On admission (T: 5 h)
Hemoglobin (g/L)	135–168	108	91
Leukocytes (10^9^/L)	4–10	372.3	384.6
Erythrocytes (10^12^/L)	4.5–5.5	3.5	3
Platelets (g/L)	150–410	226	251
International normalized ratio (INR)	0.7–1.2	1.3	1.3
C-reactive protein (CRP) (mg/L)	0–5	94.3	–
